# Lifetime Autism Spectrum Features in a Patient with a Psychotic Mixed Episode Who Attempted Suicide

**DOI:** 10.1155/2014/459524

**Published:** 2014-10-01

**Authors:** Marly Simoncini, Mario Miniati, Federica Vanelli, Antonio Callari, Giulia Vannucchi, Mauro Mauri, Liliana Dell'Osso

**Affiliations:** ^1^Section of Psychiatry, Department of Clinical and Experimental Medicine, University of Pisa, Via Roma 57, 56100 Pisa, Italy; ^2^Bipolar Disorders Program, Clinical Institute of Neuroscience, Hospital Clinic, University of Barcelona, 170 Villarroel Street, 08036 Barcelona, Catalonia, Spain

## Abstract

We present a case report of a young man who attempted suicide during a mixed episode with psychotic symptoms. The patient's history revealed the lifetime presence of signs and features belonging to the autism spectrum realm that had been completely overlooked. We believe that this case is representative of an important and barely researched topic: what happens to children with nondiagnosed and nontreated subthreshold forms of autism when they grow old. The issue of early recognition of autism spectrum signs and symptoms is discussed, raising questions on the diagnostic boundaries between autism and childhood onset psychotic spectrums among patients who subsequently develop a full-blown psychotic disorder.

## 1. Introduction

Autism spectrum disorder (ASD) encompasses a number of neurodevelopmental disorders of increasing prevalence, currently reported to affect 1 in 88 individuals [1]. Children with ASD may show signs and symptoms along a wide range of clinical severity: from social difficulties to severe functional impairment, with social communication deficits and restricted or repetitive interests and behaviors. These conditions typically present in the first 3 years of life. However, in their subtle forms they might run overlooked until adolescence or adulthood. Adult subjects with ASD sometimes display bizarre ideas, seemingly disorganized speech or unusual cognitive processes, and relevant mood swings.

The diagnosis of an adult with signs and symptoms belonging to ASD presents a number of challenges, mainly deriving from the difficulties in obtaining an accurate developmental history. Very little has been published regarding ASD diagnosis among adults. In a recent overview paper, the issue of aging with autism spectrum has been discussed, with special attention devoted to the need to gather information from available relatives of the individual with ASD [2]. The majority of the observations derive from long-term follow-up studies of children diagnosed with autism or Asperger syndrome [2]. As far as we know, the descriptions of patients referring for the first time to psychiatric services in adulthood are very limited, because of other Axis I disorders sharing features with ASD. We describe the case of a young man who attempted suicide during a mixed episode with psychotic symptoms. This was his first contact with psychiatric services. The patient's history revealed the lifetime presence of an autism spectrum disorder.

## 2. Case Presentation

Mr. A. is a 23-year-old man who attempted suicide, jumping from a second floor during his first psychiatric visit in a Public Health Psychiatric Service. He reported several fractures, including a severe spinal fracture. Apparently, the suicide attempt was related to a stressful life event (SLE), namely, “*the end of a romantic relationship*” occurring two weeks before, as reported by the patient's mother. After the SLE, Mr. A. had a two-week period characterized by a subjective change in energy levels. The patient became hyperactive, more assertive, and irritable, with constantly high levels of energy, decreased need for sleep, and excessive/inappropriate sex drive. During the last two days before the suicide attempt, the patient developed subtotal insomnia, with guilt, flight of ideas, persecutory delusions, and auditory hallucinations. The worsening of symptoms led his mother to make an appointment in an outpatient psychiatry service. During the visit, he attempted suicide, with an impulsive modality. He was hospitalized in the intensive care unit, evaluated by the liaison psychiatrist, and then moved to the Psychiatric Clinic. On the mental examination at admission he was alert and cooperative, easily irritable, and oriented in time and space, with attention procurable but difficult to maintain. The neuroimaging (MRI) performed when he was hospitalized in the intensive care unit did not provide any substantial evidence.

The patient met the DSM-IV [3] diagnostic criteria for a mixed episode with psychotic symptoms. He was treated with an atypical antipsychotic (clozapine 100 mg/daily) combined with an antiepileptic drug (valproate 300 mg/daily) and lithium (600 mg/daily). Psychotic symptoms improved. However, the staff of the inpatients section noticed a number of bizarre and eccentric behaviors. First of all, he had limited use of expressive language. He typically spoke in brief sentences and word groupings. Moreover, he appeared generally disinterested in other people, including relatives, although he seemed to tolerate their company fairly well. He therefore required his mother's supervision in eating: he ate only a limited number of foods that were accurately chosen according to color (white) and form (squared).

The previous lifetime history of the patient was negative for psychiatric interventions. However, in a more accurate lifetime assessment, including several talks with his mother, a number of relevant signs and symptoms emerged that had been completely overlooked.

Mr. A.'s mother reported that, as an infant and toddler, he was resistant to nursing and an extremely picky eater. He was difficult to settle; the only ways to manage his behaviors, which included kicking and yelling when he was distressed, were rocking him slowly and rhythmically and keeping the environment and routines consistent. The patient produced his first words around the age of 18 months. He began to combine words into sentences around the age of two. He was very particular about the foods he would eat (namely, only foods with white color or squared shape, not mixed together), and he liked to play alone for hours with a few toys. His preferred toys were squared and cold tools that he was used to take to bed with him at bedtime.

During his elementary school years, he refused to engage in classroom activities and was scarcely interested in socializing with other young children or in improving his play skills. He did not have any apparent reaction when his sister was born. However, he was most calm and content when he was at home with his grandparents, very quiet and patient persons, who lived in the suburbia. He continued with selecting his food (white and squared foods only) and drinking milk only from his infant feeding bottle, until he was an adolescent. His speech patterns were stilted and overprecise. Because his understanding of language was often literal, he frequently missed the points of other people's jokes.

During middle school, he developed friendship only with one or two boys in his class, mainly due to a mutual interest in playing video games and listening to house music. From the age of fourteen onwards, Mr. A. experienced a number of adaptive, social, and academic challenges, so his parents withdrew him several times from school. In the following years, he did not participate in work exploration programs, spending most of his time playing videogames and listening to his favorite disk jockey's house music.

Despite the signs and symptoms described, Mr. A.'s parents did not ever ask for a specialist evaluation. Mr. A.'s mother spent most of her time nurturing and caring for him, especially after his father died. She wanted to be supportive of him and encourage him to pursue his own life; at the same time, she was concerned about his work abilities and worried about any new relationship because of his difficulties in community, work, and social situations.

Over the last few years, Mr. A. seemed to improve in social skills, increasing participation with a few friends in the community and showing, for the first time, a romantic interest in a young girl. However, this relationship ended and during the last weeks Mr. A. experienced the already described symptoms.

Considering the developmental history, Mr. A. was evaluated during hospitalization by a senior psychiatrist, specialized in autism spectrum disorders, with the Autism Diagnostic Observation Schedule (ADOS) Module 4 [4], the Ritvo Autism Asperger Diagnostic Scale-Revised (RAADS-r) [5], and the Autism Diagnostic Interview-Revised (ADI-R) with his mother [6]. The ADOS is a standardized instrument that assesses social interaction, communication, and imagination during a semistructured interview with a trained examiner. Module 4 was developed for adolescents and adults with fluent speech. In the original paper on the ADOS results indicate that module 4 can be used to distinguish between spectrum and nonspectrum autism and to a lesser degree between ASD and childhood onset schizophrenia spectrum disorders. The RAADS-r was specifically designed to address a major gap in screening services for adults with autism spectrum disorders. The ADI-R is useful for diagnosing autism, planning treatment, and distinguishing autism from other developmental disorders. It provides categorical results for three domains: language/communication, reciprocal social interactions, and repetitive behaviors/interests.

No IQ examination was performed. 

Mr. A. score was as follows with the ADOS module 4:

1. communication: 2 (autism spectrum cut-off ≥2);
2. social interaction: 6 (autism spectrum cut-off ≥4);
3. imagination/creativity: 2;
4. stereotyped behaviors and restricted interests: 1.

The RAADS-r total score was 23 (negative; cut-off for the Asperger Syndrome ≥65). 

The ADI-r scored as follows:

1. language/communication: 11 (cut-off ≥8);
2. reciprocal social interactions: 10 (cut-off ≥10);
3. repetitive behaviors/interests: 6 (cut-off ≥3).

The patient was described as cooperative, even if the communication was in response to direct questions. He appeared uncomfortable engaging in imaginary play independently from the examiner. When encouraged, Mr. A. expressed his difficulties in developing friendships, especially during elementary and middle school. He described objects in a very detailed manner.

In summary, the clinical evaluation and the scoring tests suggested that an autism spectrum disorder was present even if never “suspected” before.

## 3. Discussion

Psychotic symptoms brought Mr. A. to the psychiatrist's attention for the first time, although the “triad” of ASD had actually been present from infancy (social abnormalities, communication impairments, and rigid/repetitive behaviors). However, even when present, ASD symptoms might run largely neglected in adulthood psychiatry. Many adult and older subjects with ASD remain undiagnosed and thus are largely unknown to specialist services. They might have spent childhood and adulthood by either being supported by their family or holding jobs in protected environments, enabling them to function almost “normally,” thus escaping the ASD diagnosis for the overall lifetime. In support for this consideration are the three recent case series and case reports on diagnosing adult people with ASD [7–9], indicating that the standard clinical screenings used in childhood ought to be modified and adapted for first-time diagnosis of ASD in older individuals. Moreover, the above-mentioned signs and symptoms are sometimes considered part of a “psychotic continuum,” in which early manifestations might arise in childhood, as precursors or prodromals of a subsequent full-blown psychotic disorder. However, according to the most recent epidemiological and clinical studies, childhood onset schizophrenia (COS) is a relatively rare disorder, affecting 1 in 10,000–30,000 children [2]. The diagnostic criteria are the same as those for adult onset schizophrenia, including the presence of positive and/or negative symptoms, but with onset occurring prior to the 13th birthday. COS typically presents with psychotic symptoms after the age of seven and is associated with a more severe course and poorer outcomes than adult onset schizophrenia [10]. At present ASD and COS are considered as separate clinical entities. Prior to the twentieth century, catatonia, social withdrawal, bizarre behavior, and/or psychosis in children were undifferentiated conditions, labeled as “hereditary insanity,” “dementia praecox,” or “developmental idiocy” [11]. The first and second editions of the Diagnostic and Statistical Manual of Mental Disorder (DSM-I and DSM-II) defined the “autistic behaviors and social withdrawal” as features of “childhood schizophrenia.” These entities were separated in DSM-III. Currently the DSM-5 allows the comorbid diagnoses of both conditions for those individuals with ASD who subsequently develop prominent delusions or hallucinations. The issue of a “splitting” versus a “lumping” organization of these two psychopathological areas is still in debate, given that a growing body of literature supports a neurodevelopmental origin of both schizophrenia and autism, with a shared genetic architecture contributing to, or precipitating, the development of both conditions [12–14]. Conversely, some have hypothesized that ASD and schizophrenia are diametrically opposed with respect to underlying pathology [15].

Our case report of a young man hospitalized for a suicide attempt during a mixed episode with psychotic symptoms presented the dilemma of a subthreshold psychotic disorder in childhood that finally reached the threshold for a full-blown episode in adulthood versus a subsiding comorbidity between psychosis and autism spectrum disorder. Thus, signs and symptoms of both a psychotic disorder and an ASD might run isolated or in clusters during the entire lifespan, often not reaching the threshold for a categorical diagnosis until adulthood. Rating scales of both psychopathological realms may help the clinician to detect symptoms and signs formerly not considered. However, clinical judgment can be informed but not substituted by the use of instruments that are based on the so-called “core features” of a single disorder.

We strongly believe that the use of specific instruments, such as the ADOS, the ADI-r, and the RAADS-r, designed to address a major gap in screening services for adults with ASD, should become part of routine clinical assessment for patients who have difficulties in social and nonsocial communication, in reciprocity, and in stereotyped behaviors. However, we are aware that every diagnostic conclusion in such a difficult field should always rely on expert clinical judgment, in line with the diagnostic criteria, and not solely on scores of standardized instruments, especially in a context with so many confounding factors (comorbidity, psychotic symptoms, and pharmacological treatments).

Although research on diagnosing ASD in adult people appears to be progressing, the most important step is to promote the awareness of ASD signs and symptoms during the entire lifespan, with a better definition of clinical phenotypes. This is a crucial point, especially when the diagnosis of ASD occurs during an acute psychiatric condition, with delusions and hallucinations. Since hallucinations and delusions are considered neither diagnostic nor associated features of ASD in the categories classification system, most patients with such symptoms receive a comorbid diagnosis of schizophrenia. Given that difficulties may derive from the use of categories with clear-cut boundaries between disorders, a dimensional approach might be more appropriate. Adult psychiatry does not emphasize these issues, with a trend to not train psychiatrists to diagnose ASD. The clinical result is that the most severe ASD cases often remain underdiagnosed in adult psychiatric settings.
